# Mass and Nutrient Content of Beans and Husks of *Coffea racemosa* and *Coffea zanguebariae* Grown in Mozambique

**DOI:** 10.3390/plants14213401

**Published:** 2025-11-06

**Authors:** Niquisse José Alberto, Larícia Olária Emerick Silva, Rafael Nunes de Almeida, Weverton Pereira Rodrigues, Augusto Jossias Zandamela, José Cochicho Ramalho, Fábio Luiz Partelli

**Affiliations:** 1 Department of Agrarian and Biological Sciences (DCAB), Federal University of Espírito Santo (UFES), São Mateus 29932-540, Brazil; niquissealberto@gmail.com (N.J.A.); lariciaemerick@gmail.com (L.O.E.S.); augustozandamela35@gmail.com (A.J.Z.); 2 Higher Polytechnic Institute of Mecuburi—ISPOME, Campus Tottoto, Nampula 3100, Mozambique; 3 Incaper Centro de Pesquisa e Desenvolvimento em Inovação Rural—Norte (CPDI Norte), Linhares 29915-140, Brazil; almeida.rna94@gmail.com; 4 Agricultural Sciences Center, State University of the Tocantina Region of Maranhão (UEMASUL), Imperatriz 65900-001, Brazil; 5 Mozambique Agricultural Research Institute (IIAM), Maputo 3658, Mozambique; 6 Forest Research Center (CEF), Associate Laboratory TERRA, School of Agriculture (ISA), University of Lisbon (ULisboa), 1349-017 Lisboa, Portugal; cochichor@mail.telepac.pt; 7 GeoBioSciences, GeoTechnologies and GeoEngineering Unit (GeoBioTec), NOVA School of Science and Technology (FCT/UNL), 2829-516 Caparica, Portugal

**Keywords:** wild species, coffee, dry matter, macronutrients, micronutrients

## Abstract

*Coffea racemosa* and *C. zanguebariae* show promising characteristics for cultivation under stress conditions. However, their potential for breeding programs requires further characterization, especially regarding fruit attributes. This study aimed to characterize the bean/husk ratio and the nutrient content in bean and husks from 22 accessions of *Coffea racemosa* and another 22 of *C. zanguebariae* cultivated in Mozambique. Ripe fruits were collected, dried, and manually peeled to evaluate the percentage of bean and husk. The nutrient content (N, P, K, Ca, Mg, S, Fe, Zn, Cu, Mn, and B) was quantified separately by standard methodology. The data were summarized in scatter plots, and differences among accessions were analyzed using Euclidean distance and grouped following the Unweighted Pair Group Method with Arithmetic Mean. On average, beans accounted for 54.4% (ranging from 34.5% to 66.5%) of the fruit mass in *C. racemosa* and 60.4% (38.8% to 81.4%) in *C. zanguebariae*. Macronutrient content in beans followed the order N > K > Mg > P > S > Ca (average N = 19.98 kg ton^−1^ of beans) in *C. racemosa* and N > K > Ca > Mg > S > P (average N = 25.42 kg ton^−1^ of beans) in *C. zanguebariae*. Micronutrient content in beans followed the order Fe > B > Mn > Cu > Zn in both species, with average Fe content of 325.8 and 473.72 g ton^−1^ of beans for *C. racemosa* and *C. zanguebariae*, respectively. No correspondence occurred between the bean and husk nutrient content. *Coffea racemosa* and *C. zanguebariae* exhibit bean proportions and nutritional profiles comparable to those of commercial species, highlighting their high potential for coffee diversification and genetic breeding. These results provide new evidence supporting the inclusion of *C. racemosa* and *C. zanguebariae* in breeding programs aimed at climate-resilient and nutritionally distinct coffee varieties.

## 1. Introduction

The most widely known and economically explored coffee species worldwide are *Coffea arabica* L. (Arabica) and *C. canephora* Pierre ex A. Froehner (Conilon and Robusta cultivars) [1,2]. Despite their importance, other *Coffea* species have received attention from research exploring new market niches and their use as a donor genetic material of alleles of interest for characteristics such as improved disease resistance and enhanced beverage quality [3,4,5]. Among the species of smaller commercial exploration, *C. racemosa* Lour and *C. zanguebariae* stand out due to their rarity and unique, exotic flavor profiles, which hold promise for specialty coffee markets. Therefore, agronomic work, including genetic breeding, with new *Coffea* species is essential to improve productivity, optimize harvesting, and develop more sustainable and diversified coffee production systems, especially in East Africa.

*C. racemosa* is native to central and southern Mozambique, eastern Zimbabwe, and northern South Africa [6]. It occurs in two ecological niches: (1) low altitude (<200 m) in coastal deciduous and native forests often associated with sandy soils and (2) low to medium elevations (200–600 m, not exceeding 780 m) in seasonal deciduous or semi-evergreen forests near rivers. *C. zanguebariae* emerged in southern Tanzania, northern Zimbabwe, and northern Mozambique. It has two ecological niches: (1) low altitude (5–100 m) in coastal deciduous and forests often associated with sandy soils and (2) elevations ranging from 100 to 380 m (not exceeding 680 m) in seasonal and semi-perennial deciduous forests near rivers or rocky outcrops or between rocks. According to modeling data, wild *C. racemosa* is cultivated predominantly in environments with an average annual temperature of 22.9 °C and average annual rainfall of 807 mm, while *C. zanguebariae* is cultivated at an average temperature of 24.8 °C and 998 mm of precipitation per year [1].

These species have important characteristics for the development of coffee crops under abiotic stress, including tolerance to high temperatures, water deficit, and rainfall seasonality, as well as early fruit (~120 days from flowering to fruit ripening) [1,7,8]. However, its potential as sources of alleles of interest in breeding programs and the characterization of other aspects of economic interest demand even more studies and scientific progress.

Abiotic stresses such as drought, heat, and nutrient deficiency can substantially reduce photosynthetic efficiency, impair fruit development, and alter nutrient allocation patterns within coffee plants. These stressors influence the accumulation of carbohydrates, proteins, and minerals in the reproductive organs, directly affecting grain filling and beverage quality [5]. Therefore, understanding how wild species such as *C. racemosa* and *C. zanguebariae* cope with stressful environments through adaptive physiological and nutritional traits is fundamental for selecting genotypes with superior resilience. This knowledge contributes to breeding programs aiming to develop climate-resilient cultivars capable of maintaining productivity and nutritional balance under adverse environmental conditions.

Among these, fruit-related traits are critical for determining coffee yield and beverage quality. Coffee fruits have the following morphological parts: pericarp (exocarp, mesocarp, and endocarp), perisperm, and endosperm, the latter of which stores tissue that contains the embryo [9]. Monitoring nutrient concentrations in the reproductive period of fruits is essential to estimate the nutritional needs and guide fertilization timing. For example, an analysis of nutrient content in fruits of conilon coffee genotypes showed that those with shorter maturation cycles accumulate dry matter more rapidly [10]. Knowledge about the levels of macro and micronutrients in beans can also guide specific nutritional management recommendations for each genotype [10]. Thus, information on the nutrient content in fruits, beans, and husks of genotypes is essential to genetically improve crops.

Crop nutritional status is influenced by multiple factors, including species, varieties, management practices, altitude, and maturation stage [11]. The absorption of minerals by coffee plants also varies in the same plant depending on the time of year, age, organs, and tissues and throughout its maturation cycle [12]. Environmental conditions, such as high temperatures, can also affect nutrient absorption [13,14]. The selected minerals in coffee beverages reflect the type of soil and the environmental conditions of cultivation. Minerals are typically more stable than vitamins or organic compounds in agricultural commodities and are more easily and affordably analyzed [15].

While previous studies characterized morphology and climate niches, little is known about nutrient allocation patterns in fruits of wild species such as *C. racemosa* and *C. zanguebariae*, which are crucial for breeding and specialty coffee markets. In this context, it is essential to deepen our understanding of the nutritional and morphological traits of wild coffee species with agronomic potential, such as *C. racemosa* and *C. zanguebariae*, in order to support their use in breeding programs and the development of more sustainable cultivation systems. Thus, this study aimed to characterize the bean/husk ratio and quantify the nutrient content in beans of several *C. racemosa* and *C. zanguebariae* accessions grown in Mozambique.

## 2. Results

### 2.1. Soil Characterization

The particle size analysis shows that the soil was classified as sandy [16] and the soil chemical and physical properties are listed in Table 1. This type of soil is characterized by high drainage capacity, low water and nutrient retention, and greater aeration. It is generally low in fertility and requires proper irrigation and fertilization management to maintain good productive conditions.

### 2.2. Bean-to-Husk Ratio Variability

**Figure 1:**
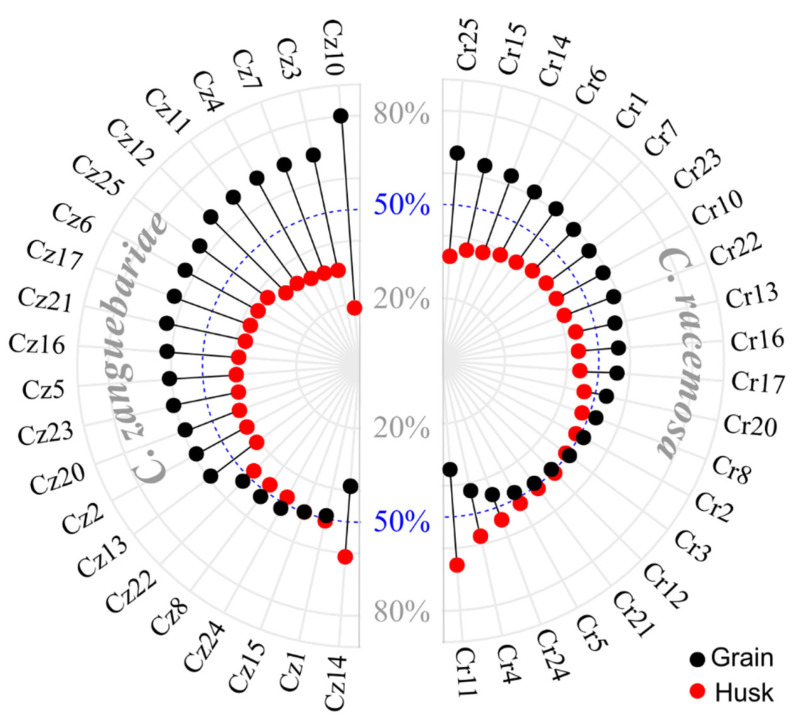
Percentage of bean and husk in the composition of fruit mass in 44 accessions of two wild species of *Coffea*: *C. racemosa* (Cr) and *C. zanguebariae* (Cz) collected in Mozambique.

The proportion of mass between beans and husks in the total fruit mass showed that, on average, 54.4% of the fruit mass in *C. racemosa* (Cr) is due to the bean, while 45.6% corresponds to the husk mass. In *C. Zanguebariae* (Cz), approximately 60.4% of the fruit mass is attributed to beans, with 39.6% corresponding to the husk mass (Figure 1). However, in *C. racemosa*, there was variation in the proportion of bean mass among the accessions, with values ranging from 34.52% (Cr11) to 66.5% (Cr25) of the total fruit mass attributed to the bean. Accessions Cr12, Cr21, Cr5, Cr24, Cr4, and Cr11 showed husk mass exceeding 50% of the total fruit mass. In contrast, accessions Cr25, Cr15, Cr14, and Cr6 presented husk mass below 40% of the total fruit mass.

Among *C. zanguebariae* accessions, variations occurred in bean mass contribution, from 38.8 (Cz14) to 81.4% (Cz10). Only accessions Cz14, Cz1, and Cz15 showed bean mass below 50% of the total fruit mass. The bean mass of Cz23, Cz5, Cz16, Cz21, Cz17, Cz6, Cz25, Cz12, Cz11, Cz4, Cz7, Cz3, and Cz10 contributed more than 60% of all total fruit mass (Figure 1).

### 2.3. Nutrient Content in Bean and Husk

**Figure 2:**
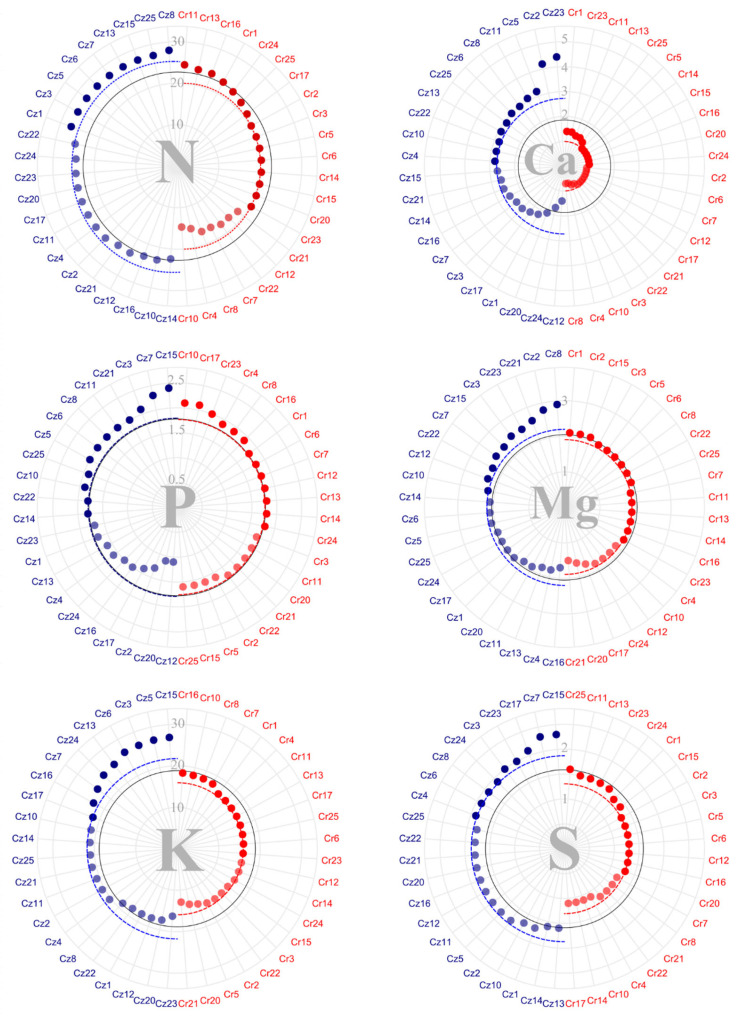
Macronutrient content (kg·ton^−1^) in bean of 22 accessions of *C. racemosa* (Cr—red) and 22 accessions of *C. zanguebariae* (Cz—blue) collected in Mozambique: nitrogen (N); phosphorus (P); potassium (K); calcium (Ca); magnesium (Mg); and sulfur (S). Continuous line = overall average; and dotted line = average of each species.

Analyses of bean macronutrient content (Figure 2) pointed to an average concentration of 19.98 kg·ton^−1^ of nitrogen (N) per bean in *C. racemosa* and 25.42 kg·ton^−1^ of N per bean in *C. zanguebariae*. Moreover, 15 *C. racemosa accessions* had an above-average N content when compared to nine *C. zanguebariae* ones. The mean content of phosphorus (P) in bean equaled 1.79 kg·ton^−1^ in *C. racemosa* and 1.76 kg·ton^−1^ in *C. zanguebariae*. In total, 13 *C. racemosa* and 12 *C. zanguebariae* accessions had P bean content above the general average. Potassium (K) content in the bean averaged 15.92 and 21.74 kg·ton^−1^ for *C. racemosa* and *C. zanguebariae*, respectively. In *C. zanguebariae*, nine accessions showed K content above average, whereas 12 *C. racemosa* ones showed above-average concentrations.

Calcium (Ca) content in the bean showed the greatest discrepancy between the species among the macronutrients, with an average of 0.99 kg·ton^−1^ in *C. racemosa* and 2.72 kg·ton^−1^ in *C. zanguebariae* (Figure 2). Notably, accessions Cz23 and Cz2 showed concentrations above 4.0 kg·ton^−1^ of Ca per bean. Regarding magnesium (Mg), the average concentration was 1.89 kg·ton^−1^ in *C. racemosa* and 2.19 kg·ton^−1^ in *C. zanguebariae*. The same accessions, Cz23 and Cz2, which stood out for their high Ca content, also showed Mg ones above the species average.

Regarding sulfur (S), *C. racemosa* showed an average concentration of 1.30 kg·ton^−1^ bean and *C. zanguebariae*, 1.86 kg·ton^−1^ (Figure 2). While nine accessions of *C. zanguebariae* had above-average S content, eight *C. racemosa* ones had below-average ones. Accessions Cz15, Cz7, and Cz17 stood out for their concentrations above 2.0 kg·ton^−1^.

Accessions Cr1, Cr13, and Cr16 showed nutrient content in the bean above the average for all macronutrients (Figure 2). Accession Cz23 showed concentrations of Ca, Mg, and S above the general average but concentrations below the average for N, P, and K. Accessions Cz3, Cz7, and Cz15 showed values above the average for all other macronutrients, except Ca. In particular, Cz15 had the highest P, K, and S content. Additionally, accession Cz8 showed the highest bean nutrient content (except for K), with the highest total for N and Mg.

**Figure 3:**
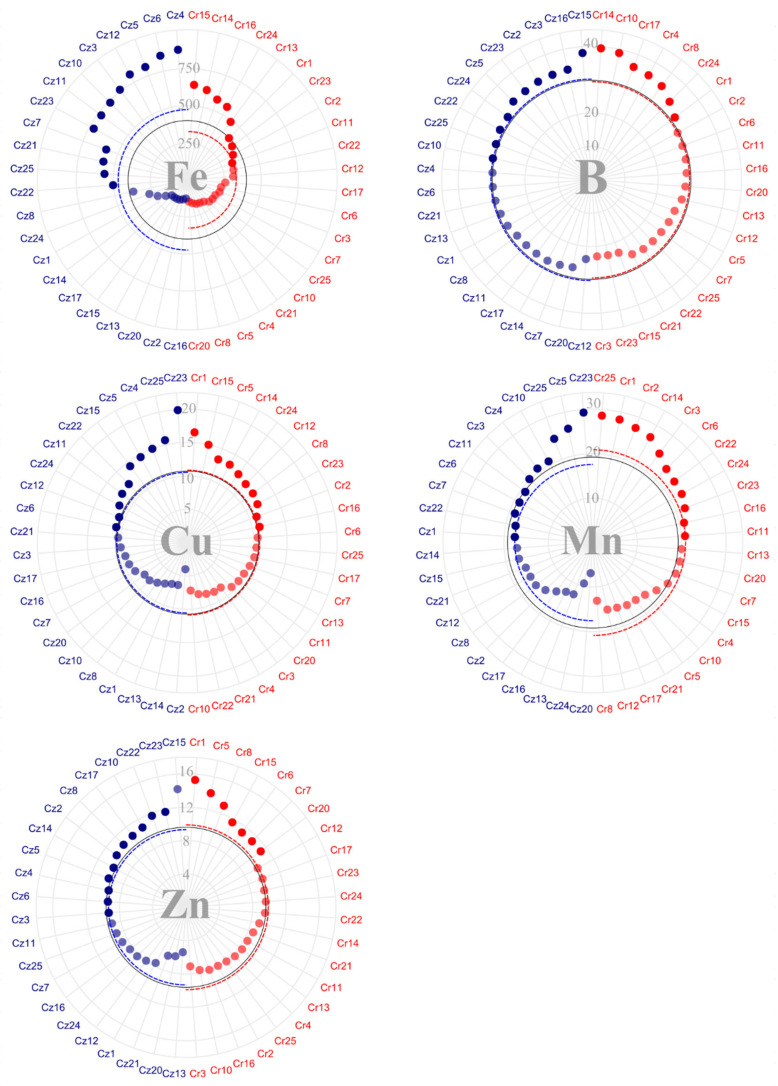
Micronutrient content (g·ton^−1^) in bean of 22 accessions of *C. racemosa* (Cr—red) and 22 accessions of *C. zanguebariae* (Cz—blue) collected in Mozambique: iron (Fe), copper (Cu), zinc (Zn), boron (B), and manganese (Mn). Continuous line = overall average; and dotted line = average of each species.

Considering micronutrients (Figure 3), iron (Fe) notably accumulated in the highest quantities in bean, averaging 325.8 g·ton^−1^ in *C. racemosa* and 473.72 g·ton^−1^ bean in *C. zanguebariae*. Cr15, Cr14, Cr16, and Cr 24 showed concentrations above 500 g·ton^−1^ of Fe and Cz4, Cz6, Cz5, Cz12, Cz3, and Cz10, concentrations above 750 g·ton^−1^.

The copper (Cu) mean bean content in the bean was 10.85 mg in *C. racemosa* and 10.60 g·ton^−1^ in *C. zanguebariae* (Figure 3). Noteworthy were accession Cz23, with a Cu content of 20 g·ton^−1^, and accession Cr1, with a concentration above 15 g·ton^−1^. In contrast, accession Cz2 showed a Cu content below 2 g·ton^−1^. For zinc (Zn), the average concentration was 9.88 g·ton^−1^ in *C. racemosa* and 9.31 g·ton^−1^ in *C. zanguebariae*. Accessions Cr1 and Cz15 stood out, with Zn content above 15 and 13 g·ton^−1^, respectively.

Boron (B) bean content showed the greatest uniformity among micro and macronutrients, averaging 29.42 g·ton^−1^ in *C. racemosa* and 30.20 g·ton^−1^ in *C. zanguebariae* (Figure 3). Concentrations near 40 g·ton^−1^ of B were found in accessions Cr14, Cr10, and Cz15. Finally, manganese (Mn) bean content totaled 20.87 g·ton^−1^ in *C. racemosa* and 17.56 g·ton^−1^ in *C. zanguebariae*. Cr25, Cr1, Cr14, Cr3, Cz23, Cz5, and Cz25 stood out with mean concentrations above 25 g·ton^−1^ (Figure 3).

Overall, regarding the micronutrient content in the bean, among *C. racemosa* accessions, Cr1 showed an above-average concentration for all micronutrients, and Cr14 and Cr24—a higher concentration for all other micronutrients, except Zn. Among the *C. zanguebariae* accessions, Cz5, Cz23, and Cz22 showed above-average concentrations for all micronutrients. Thus, considering both micro and macronutrient content in the beans, accession Cr1 stood out with consistently higher concentrations of all nutrients compared to the other *C. racemosa* accessions. In *C. zanguebariae*, however, no single accession exhibited simultaneously high concentrations for all nutrients quantified in the grains.

**Figure 4:**
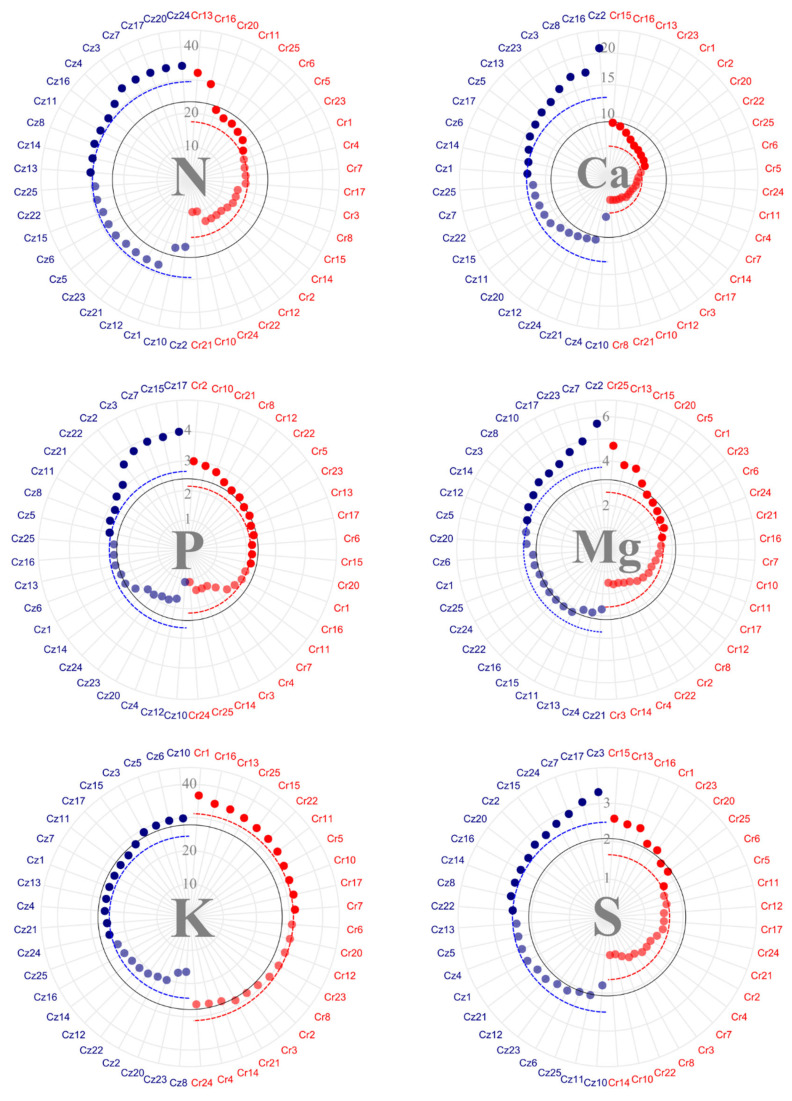
Macronutrient content (kg·ton^−1^) in the husks of 22 accessions of *C. racemosa* (Cr—red) and 22 accessions of *C. zanguebariae* (Cz—blue) collected in Mozambique: nitrogen (N); phosphorus (P); potassium (K); calcium (Ca); magnesium (Mg); and sulfur (S). Continuous line = overall average; and dotted line = average of each species.

In contrast to nutrient content in the beans, macronutrient content in the husks revealed greater discrepancies among accessions from the different species (Figure 4). N husk content was above the overall average in 11 accessions of *C. zanguebariae* and in eight accessions of *C. racemosa*. Accessions Cr13 and Cr16 stood out with the highest N content, whereas Cr21 and Cr10 showed the lowest ones out of all accessions. Regarding *C. zanguebariae* accessions, Cz10 and Cz12 stood out with lower concentrations. On average, this study found an accumulation of 17.40 kg·ton^−1^ of N in the husks of *C. racemosa* and 29.46 kg·ton^−1^ in those of *C. zanguebariae*.

The mean P content in the husks was 2.15 and 2.65 kg·ton^−1^ for *C. racemosa* and *C. zanguebariae*, respectively (Figure 4). Accessions Cr24 and Cz10 showed the lowest concentrations in their species, while accessions Cz17, Cz15, Cz7, Cz3, and Cz2 obtained values close to 4 mg of P in their husks. While the concentrations of N and P in husks were higher for *C. zanguebariae* compared to *C. racemosa*, K content showed an inverse relation. On average, there was a concentration of 31.17 kg·ton^−1^ of K in the husks of *C. racemosa* accessions, and only 24.38 kg·ton^−1^ for *C. zanguebariae* accessions. Accessions Cz8 and Cz23 stood out with lower K content in their husks.

The greater nutrient content in the husk of *C. zanguebariae* accessions compared to that of *C. racemosa* was also observed for the other macronutrients: Ca, Mg, and S (Figure 4). As with nutrient content in the beans, Ca content showed the greatest discrepancy in the husks. While *C. racemosa* accessions concentrated an average of 5.27 kg·ton^−1^ of Ca, *C. zanguebariae* showed a mean concentration of 12.99 kg·ton^−1^. A highlight for the concentration of Ca in husks refers to Cz10, with the lowest concentration in its species.

The mean concentration of Mg in the husks equaled 2.59 kg·ton^−1^ and 3.73 kg·ton^−1^ for *C. racemosa* and *C. zanguebariae*, respectively (Figure 4). Accessions Cz2 and Cz7 showed concentrations above 5 kg·ton^−1^ of Ca in their husks. Accessions Cr3, Cr14, Cr4, Cr22, and Cr2 showed values below 2 kg·ton^−1^ of Ca in their husks. As for S, the mean concentration was 1.65 in *C. racemosa* and 2.50 kg·ton^−1^ in *C. zanguebariae*, with Cz10 showing value below 2 kg·ton^−1^ of S, and Cz13 and Cz17 showing S content in the husk above 3 kg·ton^−1^.

**Figure 5:**
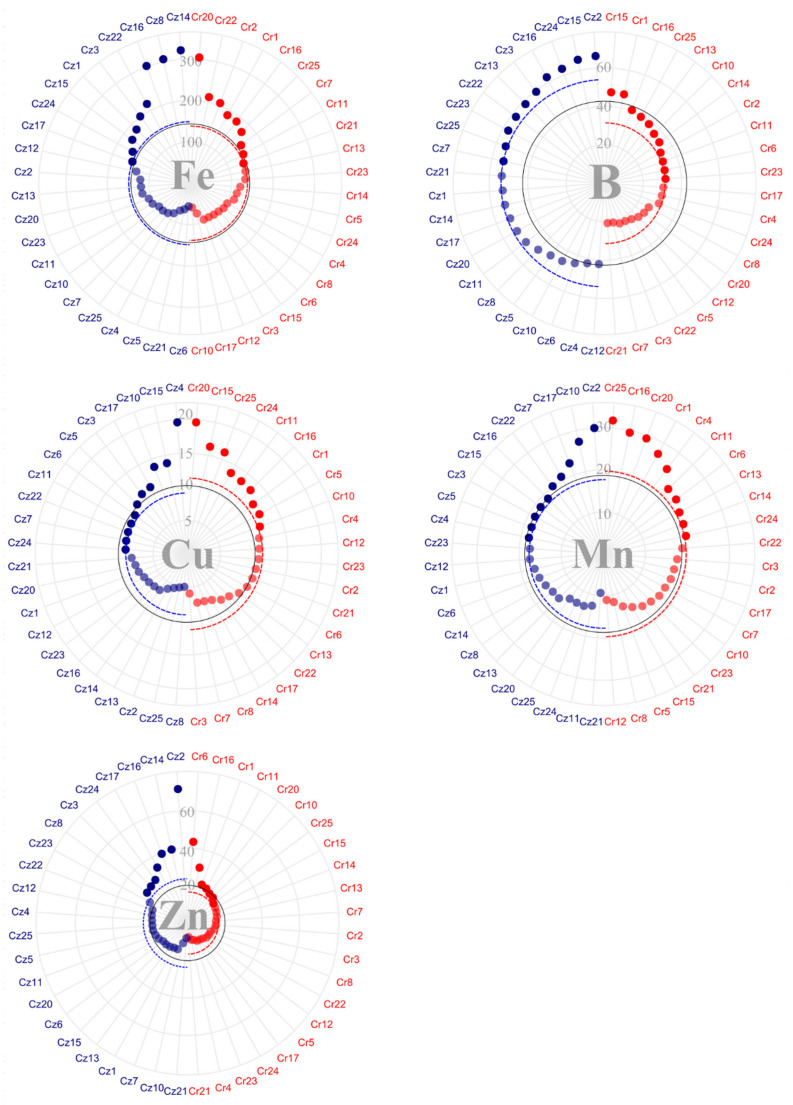
Micronutrient content (g·ton^−1^) in the husks of 22 accessions of *C. racemosa* (Cr—red) and 22 accessions *C. zanguebariae* (Cz—blue) collected in Mozambique: iron (Fe), copper (Cu), zinc (Zn), boron (B), and manganese (Mn). Continuous line = overall average and dotted line = average of each species.

Unlike bean micronutrient content, B showed the greatest discrepancy in husk concentrations (Figure 5). The concentration of Fe in the husks showed very similar averages between species (138.5 g·ton^−1^ for *C. racemosa* and 148.5 mg for *C. zanguebariae*). Despite the similarity in the averages, accessions Cr20, Cz14, Cz8, and Cz16 showed much higher husk Fe values than the other accessions (above 300 g·ton^−1^).

The *C. racemosa* accessions accumulated an average of 11.28 g·ton^−1^ of Cu in their husks, while *C. zanguebariae* accessions accumulated 9.05 g·ton^−1^. Accessions Cr20 and Cz4 accumulated close to 20 g·ton^−1^ of Cu in their husks. Mean Zn content was 16.62 and 26.63 g·ton^−1^ for *C. racemosa* and *C. zanguebariae*, respectively. Accession Cz2 showed an average 71.7 mg of Zn, while Cz14, Zz16, and Cr6, around 40 g·ton^−1^ of Zn in their husks.

Regarding B content, the greatest discrepancy occurred between species, averaging 31.41 g·ton^−1^ in *C. racemosa* and 53.89 g·ton^−1^ in *C. zanguebariae*. Accessions Cr15 and Cr1 showed values above 40 g·ton^−1^ of B in their husks, whereas Cz2, Cz15, Cz24, and Cz16, above 60 g·ton^−1^. Finally, the mean concentration of Mn equaled 19.69 g·ton^−1^ in *C. racemosa* and 17.65 in *C. zanguebariae*. Accessions Cr25, Cr16, Cr20, and Cz2 stood out with values close to or higher than 30 g·ton^−1^ of Mn in their husks.

In general, the accessions that stood out for the greater or lesser concentration of nutrients in their husks differed from those that stood out for the concentration of macro and micronutrients in their beans. A few exceptions, such as Cz2, showed higher concentrations of Ca and Mg in their husks and beans. This correlation between the husk and bean nutrient content for a few accessions and the non-correspondence of concentration for most samples showed an asymmetry between the groupings according to each part of the fruit.

### 2.4. Cluster Analysis of Accessions

**Figure 6:**
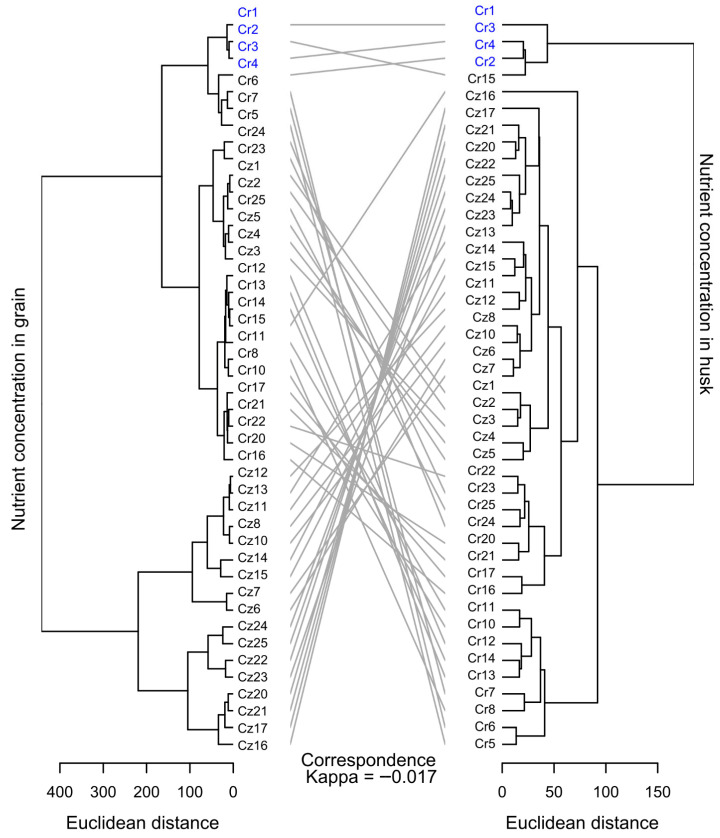
Dendrograms of dissimilarity (Euclidean distance) between 44 wild accessions of *C. racemosa* and *C. zanguebariae* for the macro and micronutrient content in their beans and husks. Lines interconnect the same accessions in the two dendrograms and express the degree of correspondence between the dissimilarity analyses. Relation by Unweighted Pair Group Method with Arithmetic Mean.

The grouping using the Unweighted Pair Group Method with Arithmetic Mean (UPGMA) considering the husk and bean nutrient content separately, showed correspondence in the formation of only one group (containing Cr1, Cr2, Cr3, and Cr4) (Figure 6). However, for husk nutrient content, the group included Cr15, unlike the grouping of accessions according to their bean nutrient content, in which Cr15 showed more than 150 units of Euclidean distance from the group above. Also, while in the concentration of nutrients in the beans, the group formed by Cr1, Cr2, Cr3, and Cr4 resembles other groups (such as that formed by Cr6, Cr7, Cr5, and Cr24), the group formed following husk nutrient content isolated other accessions by a distance greater than 150 units from Euclidean distance.

The maximum Euclidean distance between the accessions was greater when considering bean nutrient content (>400 units) in relation to the distance between accessions following husk nutrient content (<200 units). The estimated Kappa correspondence coefficient was 0.017 (Figure 6), showing the low correlation between accession groupings considering nutrient content in each part of their fruit.

Despite the lack of correspondence of distance between groups and nutrient content in the different parts of the fruit, a similarity occurred in the composition of some subgroups, such as those formed by accessions Cz1 and Cz2, by Cr23 and Cr24 and those formed by Cz1, Cz2, Cz3, Cz4, and Cz5, in which the inclusion of one or the other accession into these groups depended on the part of the analyzed fruit.

## 3. Discussion

This study evaluated the proportion between bean and husk and the nutrient content in both bean and husk of 44 wild *Coffea* accessions, showing the variations among them (Figure 1). Such variation suggests the possibility of selecting superior plants for bean production in greater proportion and in the highest concentration of nutrients in bean to the detriment of such concentrations in husks. Despite variation among accessions being evident, nutrient content in coffee fruits depends on factors such as variety, soil nutrient levels and availability, water availability, and level of shading [17,18]. Therefore, studies with stricter control of environmental factors, such as trials conducted in uniform environments and with a larger number of plants per accession, could better clarify the effectiveness of selection strategies for these characteristics [19].

The observed differences in nutrient content between accessions and between fruit parts can be explained by physiological and environmental interactions that regulate nutrient uptake, translocation, and partitioning within the plant. Under conditions of limited water availability and high temperature, common in the native habitats of *C. racemosa* and *C. zanguebariae*, plants often activate adaptive mechanisms that alter nutrient transport efficiency and storage patterns. For instance, stress conditions tend to enhance the allocation of nutrients such as calcium and magnesium to protective tissues like the husk, which play roles in maintaining cell wall integrity and osmotic balance. Conversely, higher content of nitrogen and potassium in bean metabolism contributes to grain filling and final beverage quality. Therefore, the nutrient distribution patterns observed in this study likely reflect evolutionary adaptations of these wild species to withstand environmental stress, while still maintaining fruit productivity and quality potential.

The ratio between bean and husk in this study showed that bean contributed 54.5% to the total fruit weight of *C. racemosa* and 60.4% in *C. zanguebariae*. In a study evaluating bean-to-husk weight in eight genotypes of *C. canephora*, it was found that beans accounted for approximately 50% to 70% of the fruit weight [20]. In the present study, *C. racemosa* accessions showed bean weight contributions ranging from 30% to 65%, while *C. zanguebariae* ranged from 40% to 80%. This variation shows that although these wild species remain scarcely explored, their accessions offer yield potential in a proportion compatible with consolidated commercial species, such as *C. canephora* and *C. arabica*. In addition to the commercialization potential of these beans, coffee husks can offer a useful alternative for the development of value-added products, given their physicochemical and structural characteristics, which may support applications in areas such as packaging, cosmetics, or agricultural inputs [21,22,23]. The dynamics of dry matter (DM) accumulation in the main coffee berry components (beans and husks), as well as the proportion of bean DM during the maturation phase, can be used to predict the optimal harvest time to ensure high-quality beans [24].

Regarding nutrient content in beans and husks, higher levels in beans are desirable, as this is the part of the fruit used for processing and commercialization. In general, the macronutrients of P, K, Ca, Mg, and S content were higher in the husks of *C. racemosa* than in their bean. Meanwhile, N, P, Ca, Mg, and S content were higher in the husks of *C. zanguebariae* than in their bean (Figure 2 and Figure 4). Ca and Mg content showed the greatest discrepancy between husks and fruits in both species. The literature has reported the higher abundance of Ca in the husks of coffee fruits [25,26].

Among micronutrients, B and Zn content were notably higher in the husks compared to the bean. The high amount of Zn is an expected characteristic due to the chemical nature of coffee husks, which has been widely used as an adsorbent material for capturing heavy metals in aqueous environments [27,28]. The literature had no information on the higher concentration of B in husks.

Quantifying nutrient content in fruits, husks, and beans is essential for fertilization practices, such as using husks to improve soil quality and nutrient cycling, and to update the replacement of nutrients that have been removed by harvesting and pruning [10]. Studies have shown that coffee husks accumulate large quantities of nutrients, especially N, P, K, Ca, and Mg [29], making nutrient cycling management essential for plant development [30].

Among macronutrients, beans showed higher levels of N and K and a considerable amount of Mg. A review of the nutritional potential of coffee as a beverage showed a consensus in the literature regarding its ability to provide Mg to the human diet [31]. Similarly, beans showed a considerable content of Zn, whose concentration, as per [32], tends to favor higher beverage quality in *C. arabica*. Since this factor is a universal event for the genus, *Coffea* accessions such as Cr1 and Cz15 could improve the quality of the final beverage. However, the correlation of Zn content and beverage quality in *C. racemosa* and *C. zanguebariae* still requires specific studies.

Regarding micronutrients, the evaluation of B application on the development of *C. arabica* plants showed that higher concentrations are associated with the plant’s ability to respond to abiotic stresses and inhibit the production of compounds that negatively affect the final beverage quality, such as unsaturated fats and caffeine [33]. If these findings apply to *C. racemosa* and *C. zanguebariae*, the accessions identified in this study as having high B levels may show a strong capacity to withstand stress and inhibit excessive caffeine production. These hypotheses, however, require validation through species-specific studies.

The patterns of nutrient distribution observed in *C. racemosa* and *C. zanguebariae* provide valuable insights into the adaptive strategies of wild coffee species under environmental stress. Their ability to maintain high nutrient content in the beans, even under naturally restrictive conditions such as sandy soils and irregular rainfall, suggests efficient physiological mechanisms of nutrient acquisition and partitioning. These traits, coupled with their tolerance to heat and drought, indicate that these species harbor genes of agronomic interest for improving nutrient use efficiency, yield stability, and quality in cultivated coffee. Therefore, understanding these nutrient allocation mechanisms not only broadens the ecological knowledge of wild *Coffea* species but also opens opportunities for their strategic use in breeding programs targeting climate-resilient and nutritionally superior cultivars.

Beyond their adaptive and agronomic relevance, *C. racemosa* and *C. zanguebariae* hold remarkable potential for diversifying and enriching the global coffee market. Their unique nutrient profiles and demonstrated tolerance to environmental stressors make them promising candidates for the development of high-quality, climate-resilient coffees with distinctive sensory and nutritional attributes. However, to fully harness this potential, further multidisciplinary research is needed. Future studies should include detailed sensory and chemical analyses to characterize flavor compounds, compositional assessments to link nutrient content with beverage quality, and agronomic trials to evaluate yield performance under different cultivation systems. Additionally, investigations into health-promoting properties and bioactive compounds could strengthen the scientific basis for the eventual authorization, commercialization, and market differentiation of these wild species. Such studies will be crucial to bridge the gap between genetic diversity conservation and tangible innovations in the coffee value chain.

Beans with different nutrient contents can yield beverages with distinct flavor profiles and nuances [34]. Exploring variations in these nutrient contents may help identify genetic materials more likely to express specific flavors. Moreover, managing agronomic strategies that alter the chemical composition of the beans may become a key tool in tailoring beverage quality in targeted production batches [10]. Another important finding of this study was the lack of correlation between nutrient content in beans and husks, as demonstrated by the low correspondence coefficient between the clustering of accessions based on nutrient content in different fruit parts (Figure 6). Since the primary interest of the consumer market lies in a higher concentration of nutrients in beans to the detriment of the lower concentration of nutrients in the husks of coffee fruits, positive correlations between these traits would be undesirable in breeding programs. Conversely, the absence of correlation suggests that the genes involved in nutrient accumulation in beans may act independently of those in the husks. Furthermore, the absence of strong negative or positive correlations indicates that these genes have no close links. These observations support the hypothesis that genetic improvement to increase the nutrient content in the bean will fail to directly affect the potential of genotypes to accumulate nutrients in the husk. Studies to validate this and other hypotheses in this study will contribute to the use and positioning *C. racemosa* and *C. zanguebariae* accessions in the crossbreeding blocks within the coffee genetic breeding programs.

## 4. Materials and Methods

### 4.1. Brief Characterization of Plant Material and Experimental Area

From 2023 to 2024, randomly distributed samples of commercially cultivated wild populations were collected at their cultivation sites in southern Mozambique for *C. racemosa* in Chidenguele (Gaza province), Murumbene, Maxixi, Inharrime, Panda, and Zavala (all from Inhambane province), and in northeastern Mozambique for *C. zanguebariae* in Ibo Island (Cabo Delgado province), with the specific coordinates identified at the collection sites given in Table 2. Specimens from the National Herbarium were also searched at the Institute of Agrarian Research of Mozambique (created in 1967) to obtain additional information on habitat, vegetation, environment, morphology, and cultivation. The soil sampling and physical analyses were conducted in accordance with the procedures described in [16], whereas the chemical analyses were performed by a reputable and accredited private laboratory in Brazil.

### 4.2. Fruit Collection and Nutrient Content in the Bean and Husks

The bean samples from *C. racemosa* accessions were collected in December 2023 and *C. zanguebariae* ones in February 2024, all of which were in their maturation phase. A sample size of 22 accessions per species were considered, totaling 44 plants. The fruits were manually collected once completely mature. About 200 g of fruits per plant were stored in identified paper bags and dried in the sun for seven days and then in an oven with forced air circulation at 60 °C (Model SMO1, Shel Lab, Cornelius, OR, USA) up to a constant mass. After drying, beans and husks were manually separated. The percentage of bean and the percentage of peel in the fruits were evaluated based on this sample.

These materials were collected and dried in Mozambique and sent to Brazil with authorization from the Ministry of Agriculture, Livestock and Food Supply by Import Permits no. 319/2021 and 320/2021 to quantify the nutritional content in the bean and husks. Nitrogen (N), phosphorus (P), potassium (K), calcium (Ca), magnesium (Mg), sulfur (S), iron (Fe), zinc (Zn), copper (Cu), manganese (Mn), and boron (B) content were obtained according to [35]. N content was determined by the Nessler colorimetric method after digestion with sulfuric acid; that of P, by molecular absorption spectrophotometry; that of K, by flame photometry; and that of S, by sulfate turbidimetry. Calcium (Ca), magnesium (Mg), iron (Fe), zinc (Zn), manganese (Mn), and copper (Cu) were quantified by atomic absorption spectrophotometry (AAS; Perkin Elmer, Model analyst 800, Norwalk, MA, USA). Boron (B) was determined by colorimetry using the Azomethine-H method [35].

### 4.3. Statistical Analysis

Bean and husk nutrient content data were summarized in scatter plots considering the 22 accessions of each species. The differences among accessions for the set of quantified nutrients were expressed via Euclidean distance, which grouped the accessions by the Unweighted Pair Group Method with Arithmetic means (UPGMA). In total, two dissimilarity dendrograms were built, one for bean and one for husk nutrient content. The degree of correspondence between the dendrograms was expressed by the Kappa coefficient. Analyses were performed and graphs were built on R software [36] based on the functions available in the packages cluster, factoextra, dendextend, ggforce and ggplot2.

## 5. Conclusions

*Coffea racemosa* and *C. zanguebariae* showed grain–bean proportions and nutrient profiles comparable to commercial species, underscoring their potential for coffee diversification and targeted breeding. In the beans, macronutrient concentrations followed N > K > Mg > P > S > Ca in *C. racemosa* and N > K > Ca > Mg > S > P in *C. zanguebariae*. For both species, micronutrients followed Fe > B > Mn > Cu > Zn. Notably, bean nutrient concentrations did not mirror those in the husks, indicating distinct partitioning between tissues.

Among the two species, *C. racemosa* stood out for its comparatively higher macronutrient levels (notably N and Mg), suggesting superior bean nutritional richness. Coupled with the genetic divergence observed between these wild taxa and commercial *Coffea*, our findings highlight *C. racemosa* as a prime candidate for further studies and incorporation into breeding programs aimed at improving bean quality, resilience, and by-product valorization, while *C. zanguebariae* remains a promising complementary resource.

## Figures and Tables

**Table 1:** Chemical and granulometric characteristics of the soil of the local area Cabo Delgado, Gaza and Inhambane, Mozambique.

Chemical Properties		Results				
		Unity	Ibo Irland	Chidenguele	Zavala	Panda
M.O.	Organic Matter (Oxy-Red.)	mg dm^−3^	2.5	0.9	2.6	1.2
pH (H_2_O)	(Water—Proportion 1:2.5)	unit	7.4	6.4	6.9	6.6
P	(Phosphorus)	mg dm^−3^	857.7	48.2	98.8	34.7
K	(Potassium)	mg dm^−3^	113	60	58	109
Ca	(Calcium)	cmolc dm^−3^	14.5	2.1	6.2	2.2
Mg	(Magnesium)	cmolc dm^−3^	1.3	0.5	1.3	0.8
Al	(Aluminum)	cmolc dm^−3^	0	0	0	0
H + Al	(Potential soil acidity)	cmolc dm^−3^	0	0.3	0.5	0.9
S.B.	(Sum of bases)	cmolc dm^−3^	16.1	2.8	7.7	3.3
C.T.C.	(Cation exchange capacity at pH = 7)	cmolc dm^−3^	16.1	3	8.1	4.2
V	(Base saturation)	%	100	90.2	93.9	78.5
K C.T.C	(K at Cation exchange capacity)	%	1.8	5	1.8	6.7
Ca C.T.C	(Ca at Cation exchange capacity)	%	90.1	68.8	76.1	52.6
Mg C.T.C.	(Mg at Cation exchange capacity)	%	8.1	16.4	15.9	19.1
Al C.T.C.	(Al at Cation exchange capacity)	%	0	9.8	6.1	21.5
H + Al C.T.C.	(H + Al at Cation exchange capacity)	%	0	9.8	6.1	21.5
P (Resin)		mg dm^−3^				
P-rem	(Remaining phosphorus)	mg L	29	40.6	42.5	44.5
Na	(Natrium)	mg dm^−3^	47.3	10.3	16.7	9
S	(Sulfur)	mg dm^−3^	18.5	17.5	19	18.2
B	(Boron)	mg dm^−3^	1.1	0.2	0.6	0.3
Zn	(Zinc)	mg dm^−3^	35.1	2.9	8.5	16.9
Mn	(Manganese)	mg dm^−3^	21.2	48.3	67	90.5
Cu	(Cuprum)	mg dm^−3^	0.2	0.1	0.3	0.8
Fe	(Iron)	mg dm^−3^	2.4	7.2	8.5	9.6
**Granulometric fractions (g kg^−1^)**						
Clay			123	48	98	73
Silt			11	10	15	10
Sand			866	942	887	917
Soil type	Sand					

**Table 2:** Localization of the collected accessions of *C. racemosa* (Cr) and *C*. *zanguebariae* (Cz) from several regions in Mozambique.

Identification	Location	Location		Coordinate	Coordinate	Altitude
	Province	District	Species	S	E	m
Cr1	Inhambane	Murumbene	*C. racemosa*	23°31′56.01504″	35°23′37.41576″	61
Cr2	Inhambane	Murumbene	*C. racemosa*	23°31′56.01504″	35°23′37.41576″	61
Cr3	Inhambane	Murumbene	*C. racemosa*	23°31′56.01504″	35°23′37.41576″	61
Cr4	Inhambane	Murumbene	*C. racemosa*	23°31′55.18048″	35°20′39.58152″	61
Cr5	Inhambane	Maxixi	*C. racemosa*	23°49′55.2148″	35°20′55.21128″	18
Cr6	Inhambane	Panda	*C. racemosa*	24°3′27.6714″	34°44′17.44764″	156
Cr7	Inhambane	Panda	*C. racemosa*	24°3′27.6714″	34°44′17.44764″	156
Cr8	Inhambane	Panda	*C. racemosa*	24°3′27.6714″	34°44′17.44764″	156
Cr10	Inhambane	Panda	*C. racemosa*	24°3′27.6714″	34°44′17.44764″	156
Cr11	Inhambane	Panda	*C. racemosa*	24°3′27.6714″	34°44′17.44764″	156
Cr12	Inhambane	Inharime	*C. racemosa*	24°28′30.55764″	35°1′18.50124″	48
Cr13	Inhambane	Inharime	*C. racemosa*	24°28′30.55764″	35°1′18.50124″	48
Cr14	Inhambane	Inharime	*C. racemosa*	24°28′30.55764″	35°1′18.50124″	48
Cr15	Inhambane	Inharime	*C. racemosa*	24°28′30.55764″	35°1′18.50124″	48
Cr16	Inhambane	Inharime	*C. racemosa*	24°28′36.43428″	35°1′17.44608″	48
Cr17	Inhambane	Inharime	*C. racemosa*	24°28′36.43428″	35°1′17.44608″	48
Cr20	Inhambane	Zavala	*C. racemosa*	24°30′23.7672″	34°59′57.51276″	30
Cr21	Inhambane	Zavala	*C. racemosa*	24°30′23.7672″	34°59′57.51276″	30
Cr22	Inhambane	Zavala	*C. racemosa*	24°30′23.7672″	34°59′57.51276″	30
Cr23	Inhambane	Zavala	*C. racemosa*	24°30′23.7672″	34°59′57.51276″	30
Cr24	Gaza	Chidenguele	*C. racemosa*	24°54′30.00276″	34°10′34.30524″	62
Cr25	Gaza	Chidenguele	*C. racemosa*	24°54′30.00276″	34°10′34.30524″	62
Cz01	Cabo Delgado	Ilha de Ibo	*C. zanguebariae*	12°20′38.11596″	40°35′26.55312″	14
Cz02	Cabo Delgado	Ilha de Ibo	*C. zanguebariae*	12°20′38.11596″	40°35′26.55312″	14
Cz03	Cabo Delgado	Ilha de Ibo	*C. zanguebariae*	12°20′38.11596″	40°35′26.55312″	14
Cz04	Cabo Delgado	Ilha de Ibo	*C. zanguebariae*	12°20′38.11596″	40°35′26.55312″	14
Cz05	Cabo Delgado	Ilha de Ibo	*C. zanguebariae*	12°20′38.11596″	40°35′26.55312″	14
Cz06	Cabo Delgado	Ilha de Ibo	*C. zanguebariae*	12°20′38.11596″	40°35′26.55312″	14
Cz07	Cabo Delgado	Ilha de Ibo	*C. zanguebariae*	12°20′38.11596″	40°35′26.55312″	14
Cz08	Cabo Delgado	Ilha de Ibo	*C. zanguebariae*	12°20′21.10908″	40°35′27.25908″	9
Cz10	Cabo Delgado	Ilha de Ibo	*C. zanguebariae*	12°20′21.10908″	40°35′27.25908″	9
Cz11	Cabo Delgado	Ilha de Ibo	*C. zanguebariae*	12°20′21.10908″	40°35′27.25908″	9
Cz12	Cabo Delgado	Ilha de Ibo	*C. zanguebariae*	12°20′52.28268″	40°35′36.91068″	14
Cz13	Cabo Delgado	Ilha de Ibo	*C. zanguebariae*	12°20′52.28268″	40°35′36.91068″	14
Cz14	Cabo Delgado	Ilha de Ibo	*C. zanguebariae*	12°20′52.28268″	40°35′36.91068″	14
Cz15	Cabo Delgado	Ilha de Ibo	*C. zanguebariae*	12°20′52.28268″	40°35′36.91068″	14
Cz16	Cabo Delgado	Ilha de Ibo	*C. zanguebariae*	12°20′52.28268″	40°35′36.91068″	14
Cz17	Cabo Delgado	Ilha de Ibo	*C. zanguebariae*	12°20′52.28268″	40°35′36.91068″	14
Cz20	Cabo Delgado	Ilha de Ibo	*C. zanguebariae*	12°20′15.62712″	40°35′4.00164″	12
Cz21	Cabo Delgado	Ilha de Ibo	*C. zanguebariae*	12°20′27.59388″	40°35′9.29292″	12
Cz22	Cabo Delgado	Ilha de Ibo	*C. zanguebariae*	12°20′28.8006″	40°35′8.18556″	12
Cz23	Cabo Delgado	Ilha de Ibo	*C. zanguebariae*	12°20′15.7938″	40°35′23.59536″	11
Cz24	Cabo Delgado	Ilha de Ibo	*C. zanguebariae*	12°20′15.7938″	40°35′23.59536″	11
Cz25	Cabo Delgado	Ilha de Ibo	*C. zanguebariae*	12°20′15.7938″	40°35′23.59536″	11

## Data Availability

The original contributions presented in this study are included in the article. Further inquiries can be directed to the corresponding authors.
